# Use of acetaminophen and risk of endometrial cancer: evidence from observational studies

**DOI:** 10.18632/oncotarget.16663

**Published:** 2017-03-29

**Authors:** Yuan-Yuan Ding, Peng Yao, Surya Verma, Zhen-Kai Han, Tao Hong, Yong-Qiang Zhu, Hong-Xi Li

**Affiliations:** ^1^ Department of Pain Management, Shengjing Hospital of China Medical University, Shenyang, China; ^2^ School of Undergraduate, China Medical University, Shenyang, China

**Keywords:** acetaminophen, endometrial cancer, meta-analysis, observational study, systematic review

## Abstract

Previous meta-analyses suggested that aspirin was associated with reduced risk of endometrial cancer. However, there has been no study comprehensively summarize the evidence of acetaminophen use and risk of endometrial cancer from observational studies. We systematically searched electronic databases (PubMed, EMBASE, Web of Science, and Cochrane Library) for relevant cohort or case-control studies up to February 28, 2017. Two independent authors performed the eligibility evaluation and data extraction. All differences were resolved by discussion. A random-effects model was applied to estimate summary relative risks (RRs) with 95% CIs. All statistical tests were two-sided. Seven observational studies including four prospective cohort studies and three case-control studies with 3874 endometrial cancer cases were included for final analysis. Compared with never use acetaminophen, ever use this drug was not associated with risk of endometrial cancer (summarized RR = 1.02; 95% CI: 0.93−1.13, *I*^2^ = 0%). Similar null association was also observed when compared the highest category of frequency/duration with never use acetaminophen (summarized RR = 0.88; 95% CI: 0.70−1.11, *I*^2^ = 15.2%). Additionally, the finding was robust in the subgroup analyses stratified by study characteristics and adjustment for potential confounders and risk factors. There was no evidence of publication bias by a visual inspection of a funnel plot and formal statistical tests. In summary, the present meta-analysis reveals no association between acetaminophen use and risk of endometrial cancer. More large scale prospective cohort studies are warranted to confirm our findings and carry out the dose-response analysis of aforementioned association.

## INTRODUCTION

Endometrial cancer (EC) is the six most commonly diagnosed cancer and the second most commonly diagnosed gynecologic cancer among females worldwide, with an estimated 0.32 million cases in 2012 [1]. Since previous studies have established a hypothesis that that greater lifetime exposure to estrogens, unopposed by progesterone, is important in the etiology of this disease [2, 3], therefore, several hormone-related factors including early menarche [4], late menopause [5], nulliparity [6], and obesity [7] have been identified as risk factors for EC.

During the past decade, experimental studies have postulated that chronic inflammation is related to endometrial carcinogenesis through several pathophysiologic pathways including elevations in cytokines, prostaglandins, and cyclooxygenase with concomitant oxidative stress, induces rapid cell division and DNA damage which increase the risk of malignancy [8, 9]. Nonsteroidal anti-inflammatory drugs (NSAIDs), such as aspirin, decreased cancer risk through inhibition of both cyclooxygenase-1 (COX-1) and COX-2 expression and subsequent prostaglandin synthesis, enhancement of cellular immune response, or induction of apoptosis [10–13]. However, as the major metabolite of phenacetin, acetaminophen (paracetamol), which does not have COX-2-inhibitory properties, is also considered carcinogenic to humans [14]. Although previous observational studies have focused on the relationship between acetaminophen use and EC risk [15–21], there has been no systematic review and meta-analysis comprehensively and quantitatively summarize the evidence of aforementioned issue.

Therefore, to help reconcile the aforementioned issue as well as to provide the most recent evidence, we decided to perform the present systematic review and meta-analysis of observational studies to investigate associations between use of acetaminophen and the risk of EC.

## RESULTS

### Search results, study characteristics, and quality assessment

**Figure 1:**
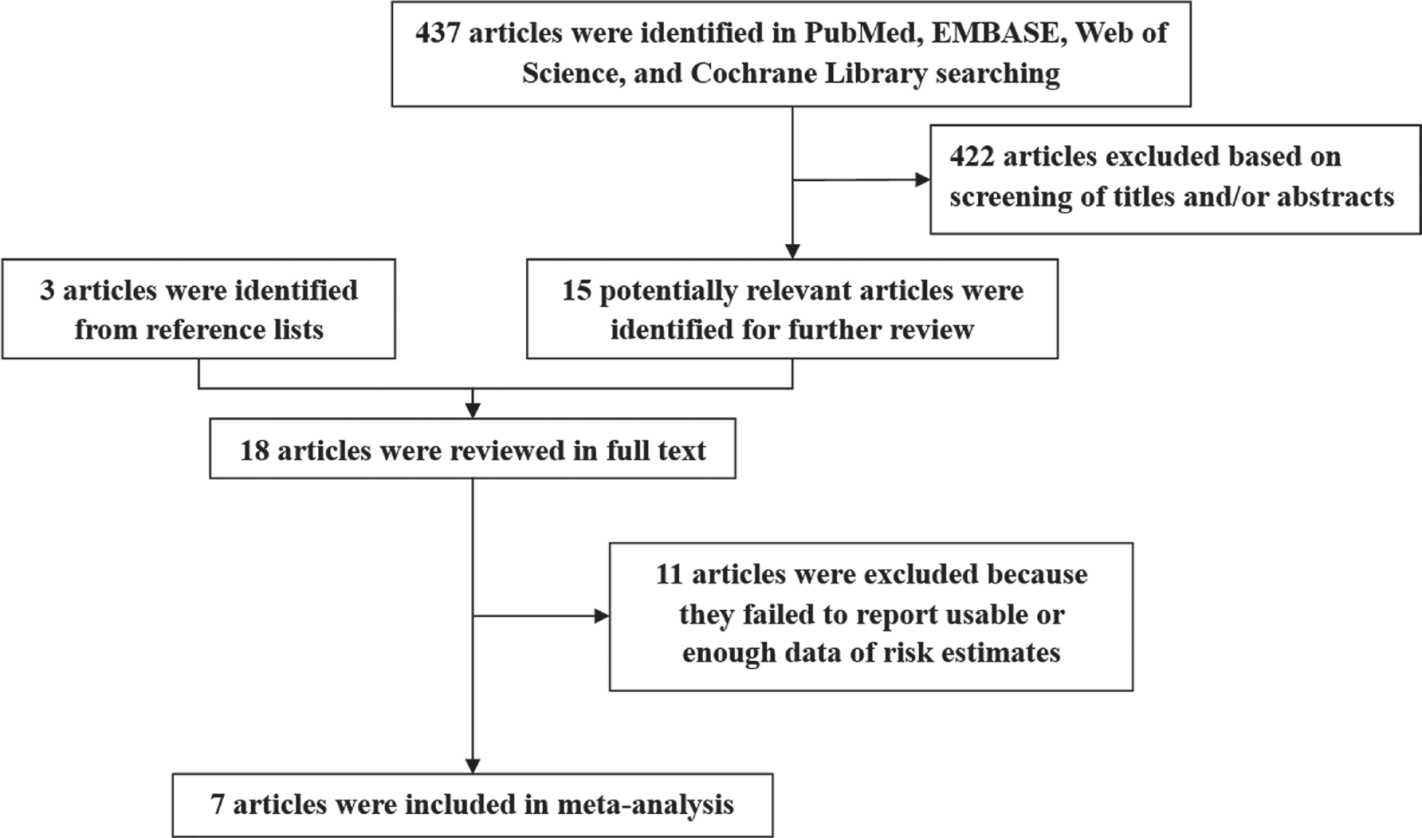
Flow diagram of the study selection for the meta-analysis

Our initial search of PubMed, EMBASE, Web of Science, and Cochrane Library databases returned 437 articles. After we screened titles and abstracts, 18 articles qualified for a full review (Figure 1). We finally included seven observational studies for the present meta-analysis [15–21].

Table 1 presents the main characteristics of the seven included studies. These studies were published from 2002 to 2013 and involved a total of 3874 EC patients with a range from 58 to 1398 cases in individual studies. Among these studies, four were prospective cohort studies [16, 17, 19, 21] and other three studies were case-control studies [15, 18, 20]. Of the four prospective cohort studies, the number of cohort participants varied from 26272 [21] to 82971 [19]. Of these three case-control studies, controls were drawn from the general population in two studies [15, 18] and hospitals in one study [20]. The majority of included studies were conducted in USA (*n* = 5), and one each was conducted in Australia [15] and Denmark [21]. All included studies adjusted for body mass index, except for study carried out by Friis et al. [21]. Five studies adjusted for age and hormone replacement therapy. However, four and three studies adjusted for parity and oral contraceptive use.

**Table 1:** Characteristics of observational studies included in the systematic review and meta-analysis

First author, [Ref] year, country	Study design	No. of case/controls (cohort)	Exposure category	Risk estimates (95% CI)	Adjustment for confounders				
					Age	BMI	Parity	HRT	OC use
Neill et al. [15], 2013, Australia	Population-based case-control	1398/740	Ever vs. Never ≥ 2 week vs. Never	1.19 (0.86−1.65) 0.77 (0.41−1.45)	√	√	√	√	√
Setiawan et al. [16], 2012, USA	Cohort	620/64000	Ever vs. Never ≥ 6 years vs. Never	0.96 (0.81−1.13) 0.80 (0.61−1.06)	√	√	√	√	√
Walter et al. [17], 2011, USA*	Cohort	214/32059	Ever vs. Never ≥4 d/week and ≥ 4 years vs. Never	1.05 (0.72−1.54) 0.99 (0.48−2.01)	√	√	×	√	×
Bodelon et al. [18], 2009, USA	Population-based case-control	410/356	Ever vs. Never ≥ 10 years vs. Never	1.11 (0.70−1.77) 1.80 (0.91−3.56)	√	√	×	√	×
Viswanathan et al. [19], 2008, USA*	Cohort	747/82971	Ever vs. Never ≥ 6–7 d/weeks vs. Never	1.11 (0.70−1.77) 0.86 (0.57−1.30)	×	√	√	√	√
Moysich et al. [20], 2005, USA	Hospital-based case-control	427/427	Ever vs. Never ≥ 10 years vs. Never	0.96 (0.60−1.54) 0.49 (0.15−1.60)	√	√	√	×	×
Friis et al. [21], 2002, Denmark	Cohort	58/26272	Ever vs. Never	1.10 (0.80−1.40)	×	×	×	×	×

BMI, body mass index; CI, confidence interval; HRT, hormone replacement therapy; N/A, not available; OC, oral contraceptive; Ref, reference.

*Risk estimates were recalculated by the method proposed by Hamling et al. [41].

According to quality assessment criteria (Supplementary Tables 1 and 2), for prospective cohort studies, the majority of included studies met the criteria except for study carried out by Friis et al. [21] which was attributed to provide crude risk estimate as well as limited follow-up time. For case-control studies, the majority of included studies met the criteria except for study carried out by Moysich et al. [20] which was attributed to hospital-based controls and significant difference in the response rate between cases and controls.

**Table 2:** Summary risk estimates of the association between acetaminophen use and endometrial cancer risk

	No. of Study	RR	95% CI	*I*^2^ (%)	*P*_h_^†^	*P*_h_^‡^
**Overall**	7	1.02	0.93–1.13	0	0.93	
**Subgroup analyses**						
**Study design**						0.48
Cohort study	4	1.00	0.90–1.12	0	0.86	
Case-control study	3	1.11	0.88–1.40	0	0.76	
**Number of cases**						0.63
≥ 450	3	1.01	0.89–1.13	0	0.52	
< 450	4	1.07	0.89–1.29	0	0.97	
**Exposure measurement**						0.61
Questionnaire	6	1.01	0.91–1.13	0	0.91	
Medication database	1	1.10	0.83–1.46	N/A	N/A	
**Geographic location**						0.32
USA	5	0.99	0.89–1.11	0	0.97	
Non-USA	2	1.14	0.92–1.41	0	0.72	
**Adjustment for risk factors**						
**Age**						0.81
Yes	5	1.01	0.89–1.15	0	0.81	
No	2	1.04	0.88–1.23	0	0.63	
**Body mass index**						0.61
Yes	6	1.01	0.91–1.13	0	0.91	
No	1	1.10	0.83–1.46	N/A	N/A	
**Parity**						0.53
Yes	4	1.00	0.89–1.13	0	0.72	
No	3	1.09	0.89–1.33	0	0.98	
**OC use**						0.63
Yes	3	1.01	0.89–1.13	0	0.52	
No	4	1.07	0.89–1.29	0	0.97	
**HRT use**						0.75
Yes	5	1.02	0.91–1.13	0	0.82	
No	2	1.06	0.84–1.35	0	0.63	

CI, confidence interval; HRT, hormone replacement therapy; N/A, not available; OC, oral contraceptive; RR, relative risk.

† *P*-value for heterogeneity within each subgroup.

‡ *P*-value for heterogeneity between subgroups with meta-regression analysis.

### Ever versus never use

**Figure 2:**
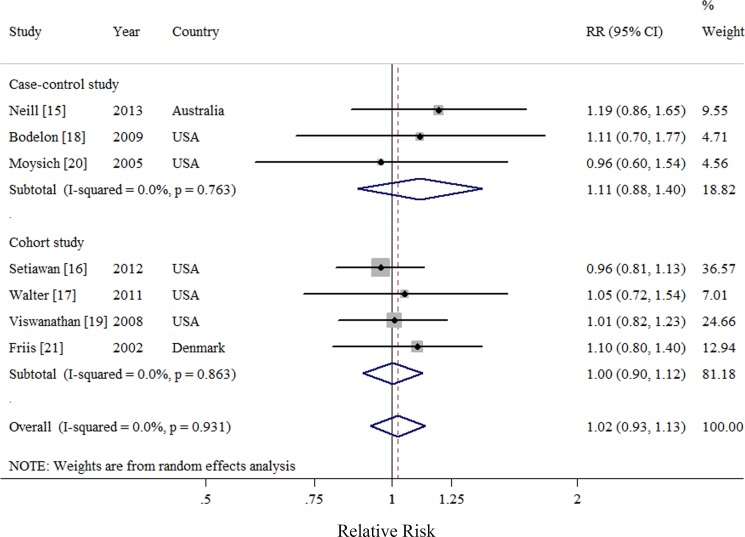
Forest plot of ever use of acetaminophen and endometrial cancer risk using random-effects model by study design The squares indicate study-specific relative risk (size of the square reflects the study specific statistical weight); the horizontal lines indicate 95%CIs; and the diamond indicates the summary relative risk estimate with its 95% CI. CI: confidence interval; RR, relative risk.

**Figure 3:**
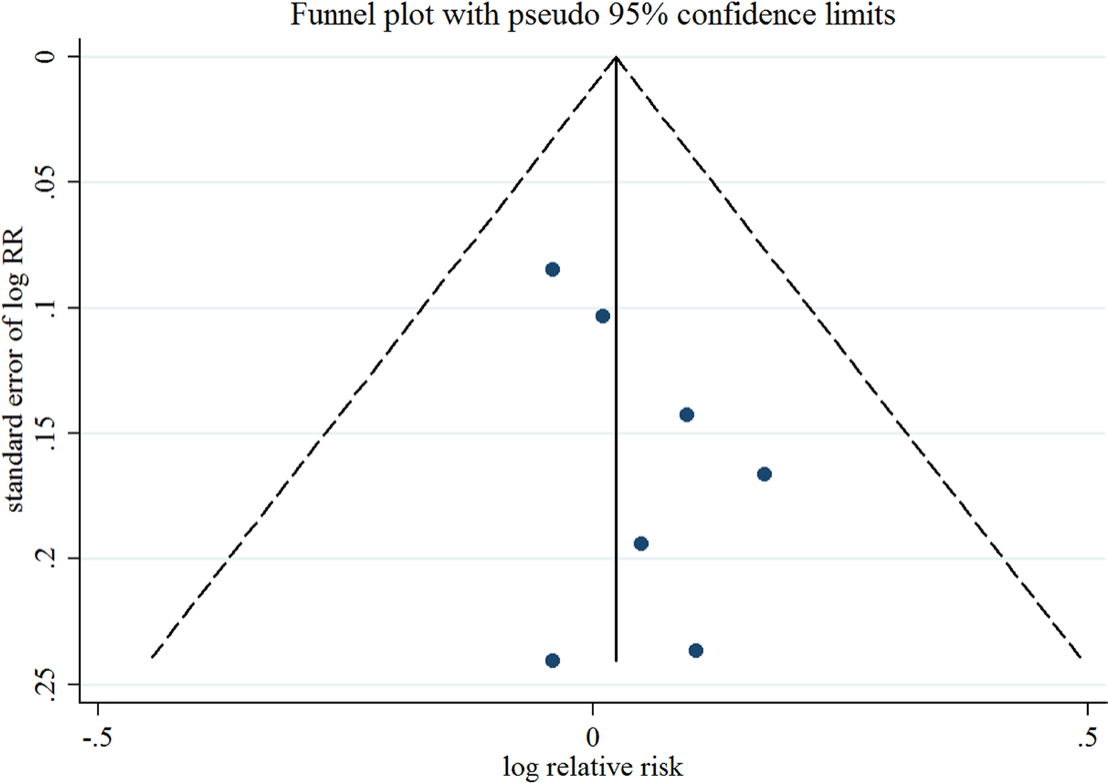
Funnel plot corresponding to the random-effects meta-analysis of the relationship between ever use of acetaminophen and endometrial cancer risk RR, relative risk.

Seven observational studies totaling 3874 EC patients evaluated the association between acetaminophen use and risk of EC [15–21]. We observed null association between ever use of acetaminophen *versus* no use and risk of EC (Summarized RR = 1.02; 95% CI: 0.93–1.13), without heterogeneity (*I*^2^ = 0%) (Figure 2). There was no evidence of publication bias by inspecting a funnel plot (Figure 3) and formal statistical tests (Egger test, *P* = 0.18; Begg test, *P* = 0.76).

Null results were observed throughout the subgroup analyses by study characteristics and adjustment for potential confounders and risk factors (Table 2). In addition, the result of meta-regression analysis suggested no evidence of significant heterogeneity between subgroups. Sensitivity analysis was performed by excluding one study at a time showed that the summarized RR ranged from 1.01 (95% CI = 0.91–1.12; *I*^2^ = 0%) when Neill et al. [15] was excluded, to 1.06 (95% CI = 0.94–1.21; *I*^2^ = 0%), when Setiawan et al. [16] was excluded.

### Highest category (frequency/duration) versus never use

**Figure 4:**
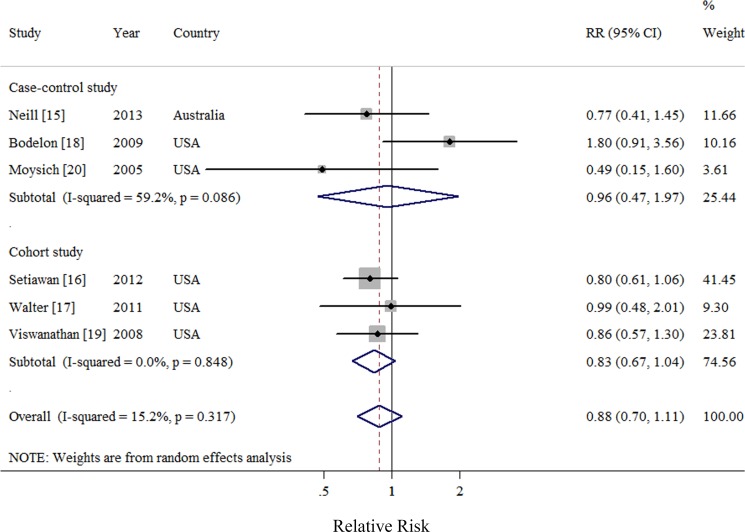
Forest plot of frequency/duration of acetaminophen use and endometrial cancer risk using random-effects model by study design The squares indicate study-specific relative risk (size of the square reflects the study specific statistical weight); the horizontal lines indicate 95% CIs; and the diamond indicates the summary relative risk estimate with its 95% CI. CI: confidence interval; RR, relative risk.

**Figure 5:**
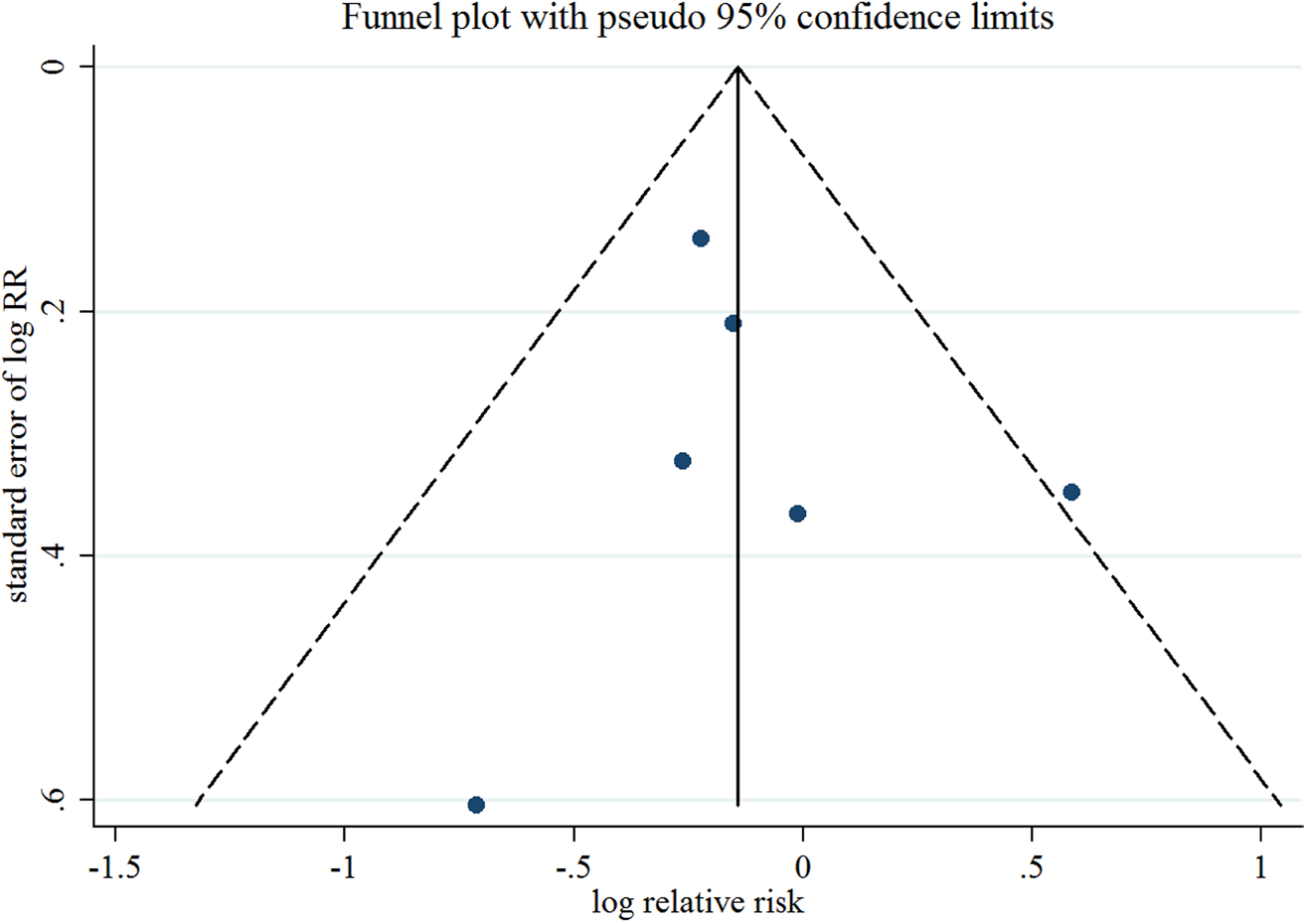
Funnel plot corresponding to the random-effects meta-analysis of the relationship between frequency/duration of acetaminophen use and endometrial cancer risk RR, relative risk.

Six observational studies totaling 3816 patients evaluated the association between acetaminophen use and risk of EC [15–20]. We observed null association between highest frequency/duration of acetaminophen using *versus* no use and risk of EC (Summarized RR = 0.88; 95% CI: 0.70–1.11), with moderate heterogeneity (*I*^2^ = 15.2%) (Figure 4). There was no evidence of publication bias by a visual inspection of a funnel plot (Figure 5) and formal statistical tests (Egger test, *P* = 0.70; Begg test, *P* = 0.85).

Negative findings were observed throughout the subgroup analyses by study characteristics and adjustment for potential confounders and risk factors (Table 3). Although the result of meta-regression analysis suggested no evidence of significant heterogeneity between subgroups, we still observed moderate heterogeneity in several subgroups. Sensitivity analysis was performed by excluding one study at a time showed that the summarized RR ranged from 0.95 (95% CI = 0.68–1.32; *I*^2^ = 23.5%) when Bodelon et al. [18] was excluded, to 0.81 (95% CI = 0.66–1.00; *I*^2^ = 0%), when Setiawan et al. [16] was excluded.

**Table 3:** Summary risk estimates of the association between frequency/duration of acetaminophen use and endometrial cancer risk (highest category versus never)

	No. of Study	RR	95% CI	*I*^2^ (%)	*P*_h_^†^	*P*_h_^‡^
**Overall**	6	0.88	0.70–1.11	15.2	0.32	
**Subgroup analyses**						
**Study design**						0.52
Cohort study	3	0.83	0.67–1.04	0	0.85	
Case-control study	3	0.96	0.47–1.97	59.2	0.09	
**Number of cases**						0.23
≥ 450	3	0.81	0.66–1.01	0	0.95	
< 450	3	1.08	0.56–2.09	47.6	0.15	
**Exposure measurement**						N/A
Questionnaire	6	0.88	0.70–1.11	15.2	0.32	
Medication database	0	N/A	N/A	N/A	N/A	
**Geographic location**						0.74
USA	5	0.91	0.69–1.21	30.3	0.22	
Non-USA	1	0.77	0.41–1.45	N/A	N/A	
**Adjustment for risk factors**						
**Age**						0.89
Yes	5	0.91	0.66–1.26	32.1	0.21	
No	1	0.86	0.57–1.30	N/A	N/A	
**Body mass index**						N/A
Yes	6	0.88	0.70–1.11	15.2	0.32	
No	0	N/A	N/A	N/A	N/A	
**Parity**						0.13
Yes	4	0.80	0.65–0.99	0	0.85	
No	2	1.35	0.75–2.42	28.8	0.24	
**OC use**						0.23
Yes	3	0.81	0.66–1.01	0	0.95	
No	3	1.08	0.56–2.09	47.6	0.15	
**HRT use**						0.44
Yes	5	0.90	0.71–1.15	19.6	0.29	
No	1	0.49	0.15–1.60	N/A	N/A	

CI, confidence interval; HRT, hormone replacement therapy; N/A, not available; OC, oral contraceptive; RR, relative risk.

† *P*-value for heterogeneity within each subgroup.

‡ *P*-value for heterogeneity between subgroups with meta-regression analysis.

## DISCUSSION

In this first meta-analysis with 3874 EC patients and 205,186 non EC populations, we found that ever use of acetaminophen was not associated with risk of EC. Additionally, more frequent or longer duration of acetaminophen use was still not associated with EC risk. These negative findings were robust in the numerous subgroup and sensitivity analyses.

Although there have been increasing evidence that inflammation and the COX pathway are involved in endometrial carcinogenesis, to some extent, the null association between acetaminophen use and EC risk is biologically plausible. NSAIDs act through inhibiting prostaglandin synthesis and may prevent carcinogenesis through COX-dependent or COX-independent mechanisms [22–24]. COX-1 is expressed constitutively, whereas COX-2 is an inducible enzyme and has been implicated in the development of cancer [23, 25]. *In vitro* studies suggested a higher COX-2 expression in EC cells compared with normal endometrium [25, 26], and inhibition of EC cell growth induced by NSAIDs [27, 28]. Of note, COX-2 selective inhibitors have been shown *in vitro* to markedly inhibit the proliferation of EC cells in a time and dose dependent manner [27, 29]. However, recently, experimental studies have found limited evidence of chemopreventive roles of acetaminophen, the major metabolite of phenacetin, does not have COX-2-inhibitory properties [14].

Experimental studies have proposed that obesity leads to several proinflammatory processes as well as increases the levels of circulating estrogens [30, 31]. Therefore, obesity might be a modifier between the acetaminophen use and EC risk. However, only two included studies carried out the subgroup analysis stratified by body mass index. Although we observed some variation in the direction and size of association in the results of subgroup analysis, none of the associations showed statistically significance. Similar patterns were observed in the stratified analysis by menopausal hormone use which is one of the established risk factors of EC [16]. Since the limited cases appeared in their primary subgroup analysis, further studies with larger sample size or pooled analysis are warranted to verify these issues.

An important strength of our study is that we first comprehensively and quantitatively summarize the evidence of acetaminophen use and EC risk on the basis of observational studies so far. Furthermore, compared with single study, the sample size of the present meta-analysis was relatively large, allowing for a careful evaluation of acetaminophen use. Notably, these findings were consistent throughout the subgroup and sensitivity analyses. Despite these strengths, several limitations merit discussion. First, our meta-analysis included both cohort and case-control studies. Compared with prospective cohort studies, case-control studies are more likely to subject to several bias including recall and selection bias. However, the findings of the present study were similar in subgroup analysis stratified by study design, though significant heterogeneity was observed in case-control studies when summarized the association between frequency/duration of acetaminophen use and EC risk. Second, since the majority of included studies utilized questionnaire to collect the acetaminophen use [15–20], misclassification of exposures might be a concern in this study which may have biased the results toward the null [16]. Of note, we found the category of acetaminophen use was different among these included studies. One study defined ‘never use’ as never use any type of NSAIDs for more than 5 days per month for at least 6 months [18]. In contrast, other study defined that variable as never use acetaminophen at least 2 times a week (for 1 month or longer) [16]. Additionally, limited studies considered the number of brands, different doses, and the complexity of the formulations of acetaminophen which make it unlikely that ascertainment of use of acetaminophen was perfectly precise [18]. Moreover, we failed to carry out dose-response analysis to quantitate the effect of one tablet per day of acetaminophen on EC risk because the aforementioned phenomenon as well as the limited available data of these included studies. Consequently, whether there is a non-linear aforementioned association has been still unknown. Third, although geographic location was not the source of heterogeneity, the point estimate was slightly different between studies carried out in USA and outside of USA. This phenomenon might be attributed to the different prevalence of acetaminophen use. For example, the prevalence of acetaminophen use was 87.8% and 88.6% in EC cases and controls, respectively, in the Australian National Endometrial Cancer Study (ANECS), a population-based case-control study [15]. However, the aforementioned prevalence was 27.1% in 32059 populations in the prospective VITamins and Lifestyle (VITAL) study which was carried out in western Washington State [17]. Finally, previous studies mentioned that use of NSAIDs including acetaminophen might be associated with body mass index, cigarette smoking, parity, age at menarche, oral contraceptive use, and menopausal hormone use [16, 32, 33]. Although not all potential confounders or important risk factors were adjusted for in every study, many but not all of the studies carried out these adjustment. In the subgroup analyses stratified by these potential confounders or important risk factors, the results of meta-regression analyses did not show significant difference between these findings. Furthermore, since quality scoring might hide important information by combining disparate study features into a single score as well as introduce an arbitrary subjective element into the analysis, therefore, we used the NOS instead of a scoring system.

In conclusion, after summarizing the results of four cohorts and three case-control studies, the present systematic review and meta-analysis failed to find any association between ever use acetaminophen as well as more frequency and longer duration of acetaminophen use and EC risk. Larger prospective cohort studies are warranted to verify our findings and carry out the dose-response analysis of aforementioned association.

## MATERIALS AND METHODS

### Data sources and searches

Following recommendations of the Meta-analysis of Observational Studies in Epidemiology group (MOOSE) [34], we systematically searched electronic databases (PubMed, EMBASE, Web of Science, and Cochrane Library) for observational studies up to February 28, 2017 using a search strategy with the following keywords: “analgesics”, “acetaminophen”, “paracetamol”, “aspirin”, “nonsteroidal anti-inflammatory agents”, or “NSAID”, and “endometrium”, or “endometrial”, and “cancer”, “tumor”, “carcinoma”, or “neoplasm”. Additionally, we manually checked reference lists to identify other potential studies.

### Study selection and exclusion

Reference manager Endnote was used to identify and remove duplicate publications [35, 36]. Subsequently, studies were included for analysis if met the following inclusion criteria: (i) observational studies investigated the association between acetaminophen/paracetamol and risk of endometrial cancer, and (ii) studies provided odds ratio (OR) or risk ratio or relative risk (RR) with 95% confidence intervals (CIs) or data necessary to calculate those risk estimates. We excluded studies if they met the following exclusion criteria: (i) reviews without original data, ecological studies, editorials, and case reports; (ii) studies reported with risk estimates that could not be summarized (e.g., studies reported without 95% CIs). If multiple articles were derived from the same study and reported the same associated events, we only included the latest published data for our primary analysis.

### Data abstraction and quality assessment

Two independently investigators (Y-YD and PY) extracted the information such as first author, year of publication, country, and number of endometrial cancer cases and controls/cohort, exposure characteristics, and study-specific adjusted risk estimates with their 95% CIs (including adjusted risk factors if applicable). All differences were resolved by a third investigator (Z-KH). Subsequently, these information was recorded on pretested standard forms. Additionally, these two investigators (Y-YD and PY) assessed the methodological quality of these included observational studies according to the Newcastle-Ottawa quality assessment scale [35–37].

### Statistical analysis

Since the low incidence of endometrial cancer, we assumed that estimates of ORs from case-control studies and risk ratio, or RRs from cohort studies were all valid estimates of the RR, and therefore, we reported all results as the RR for simplicity [6, 38–40]. For studies that did not present results for ever use *vs*. never use acetaminophen, we followed the method proposed by Hamling et al. [41] to recalculate the RR. Briefly, Hamling et al. [41] described a method for estimating the alternative comparisons when summarizing published evidence potential improving the reliability of a meta-analysis.

Random effect models were used to summarize the risk estimates of each study. Heterogeneity among studies was assessed with the *I*^2^ statistic which indicated significant heterogeneity when this value greater than 50% [42]. Subgroup analyses were conducted to study design (cohort *vs*. case-control studies), median number of endometrial cancer cases (≥ 450 *vs*. < 450), geographic location (USA *vs*. Non-USA), exposure measurement (questionnaire *vs*. medication database), and adjustments for risk factors including age, body mass index, parity, oral contraceptive use, and hormone replacement therapy use. Heterogeneity between subgroups was evaluated by meta-regression. Publication bias was assessed by inspecting a funnel plot for outcome and further tested with Begg's [43] and Egger's [44] test. Additionally, to assess the effect of individual studies on the estimated RR, we conducted a sensitivity analysis in which we recalculated the summarized RR by omitting one study at a time. All statistical analyses were performed using Stata 12.0 (StataCorp LP).

## SUPPLEMENTARY MATERIALS TABLES


