# Supplementary material for: Use of acetaminophen and risk of endometrial cancer: evidence from observational studies
Source: Oncotarget. 2017 Mar 29;8(21):34643–51. doi: 10.18632/oncotarget.16663 (PMC5470998; doi:10.18632/oncotarget.16663)
Supplement: Supplementary file 1 [file oncotarget-08-34643-s001.pdf]

Use of acetaminophen and risk of endometrial cancer: evidence from observational studies

Supplementary Materials

Supplementary Table 1: Methodological quality of cohort studies included in the systematic review and meta-analysis

First author [reference], year	Representativeness of the exposed cohort	Selection of the unexposed cohort	Ascertainment of exposure	Outcome of interest not present at start of study	Control for important factor or additional factor †	Assessment of outcome	Follow-up long enough for outcomes to occur‡	Adequacy of follow-up of cohorts§
Setiawan [16], 2012	*	*	*	*	**	*	*	*
Walter [17], 2011	*	*	*	*	**	*	-	*
Viswanathan [19], 2008	*	*	*	*	**	*	*	*
Friis [21], 2002	*	*	*	*	-	*	-	*

†A maximum of 2 stars could be awarded for this item. Studies that controlled for body mass index received one star, whereas studies that controlled for other important confounders such as parity, oral contraceptive use, hormone replacement therapy received an additional star.
‡A cohort study with a median follow-up time >10 years was assigned one star.
§A cohort study with a follow-up rate > 75% was assigned one star.

Supplementary Table 2: Methodological quality of case-control studies included in the systematic review and meta-analysis

First author [reference], year	Adequate definition of cases	Representativeness of cases	Selection of control subjects	Definition of control subjects	Control for important factor or additional factor†	Exposure assessment	Same method of ascertainment for all subjects	Non response Rate‡
Neill [15], 2013	*	*	*	*	**	*	*	-
Bodelon [18], 2009	*	*	*	*	**	*	*	*
Moysich [20], 2005	*	*	-	*	**	*	*	-

†A maximum of 2 stars could be awarded for this item. Studies that controlled for body mass index received one star, whereas studies that controlled for other important confounders such as parity, oral contraceptive use received an additional star.
‡One star was assigned if there was no significant difference in the response rate between control subjects and cases by using the chi-square test ($P > 0.05$).
